# Impact of Dimensionality and Network Disruption on Microrheology of Cancer Cells in 3D Environments

**DOI:** 10.1371/journal.pcbi.1003959

**Published:** 2014-11-20

**Authors:** Michael Mak, Roger D. Kamm, Muhammad H. Zaman

**Affiliations:** 1 Mechanical Engineering, Massachusetts Institute of Technology, Cambridge, Massachusetts, United States of America; 2 Biomedical Engineering, Boston University, Boston, Massachusetts, United States of America; University of California San Diego, United States of America

## Abstract

Dimensionality is a fundamental component that can have profound implications on the characteristics of physical systems. In cell biology, however, the majority of studies on cell physical properties, from rheology to force generation to migration, have been performed on 2D substrates, and it is not clear how a more realistic 3D environment influences cell properties. Here, we develop an integrated approach and demonstrate the combination of mitochondria-tracking microrheology, microfluidics, and Brownian dynamics simulations to explore the impact of dimensionality on intracellular mechanics and on the effects of intracellular disruption. Additionally, we consider both passive thermal and active motor-driven processes within the cell and demonstrate through modeling how active internal fluctuations are modulated via dimensionality. Our results demonstrate that metastatic breast cancer cells (MDA-MB-231) exhibit more solid-like internal motions in 3D compared to 2D, and actin network disruption via Cytochalasin D has a more pronounced effect on internal cell fluctuations in 2D. Our computational results and modeling show that motor-induced active stress fluctuations are enhanced in 2D, leading to increased local intracellular particle fluctuations and apparent fluid-like behavior.

## Author Summary

Biomechanical properties at the cellular and subcellular levels are important in providing proper biological functions, from cell migratory capabilities to intracellular transport. Deregulation in these properties can lead to disease states such as cancer metastasis. We develop and demonstrate an integrated experimental and computational approach to study intracellular mechanics. We demonstrate that a key environmental factor, dimensionality, plays a significant role in modulating intracellular mechanical behavior. This is important as typical cell biology and mechanics experiments are performed on 2D substrates, which do not capture the physiological features of 3D matrices and may not induce physiologically accurate cell properties. We further develop an effective temperature model to describe how dimensionality changes intracellular particle motion by altering the activity of molecular motors.

## Introduction

Mechanical properties of cells have important implications in many areas of biology and medicine, from cancer metastasis to blood-borne diseases to cardiovascular functions [1]–[7]. For instance, recent studies have shown that highly metastatic cancer cells tend to generate higher traction forces [1], [8] and are more deformable [9]–[13]. Understanding intrinsic intracellular mechanical properties, such as internal fluctuations and viscoelasticity, can provide insights toward fundamental functional capabilities of cells, including the abilities to migrate, change shape, and exert and respond to force. There are a number of techniques that have been developed that enables cell mechanical properties to be investigated, including micropipette aspiration [14]–[16], atomic force microscopy (AFM) [17], traction force microscopy [18], [19], optical [9] or hydrodynamic force-based cell stretching [10], and various forms of particle tracking microrheology [20]–[25]. These techniques have revealed important insights towards the mechanical states of cells. However, recent studies have shown that the cell microenvironment plays a critical role in regulating cell properties and behavior. Effects such as dimensionality, shear flow, interstitial flow, chemokine gradients, co-culture conditions, and matrix and substrate mechanics have all been demonstrated to alter cell migratory behavior, mechanical properties, and signaling [2], [26]–[40].

While cell mechanics is of considerable importance, it is currently difficult to measure mechanical properties in physiologically realistic, 3D environments. Many techniques can only be applied to cells in 2D or suspended cells. Passive particle tracking microrheology is the most practical technique for this task, as no additional constructs and instrumentation are required besides the ability to visualize intracellular particles. The microrheology technique refers to the tracking of tracer particles and assessing mechanical properties based on the particle motions. In the “passive” case, no external forces are applied and the particle motions are intrinsic to the material [41], [42]. For cells, those forces result from thermal activity and molecular motors. This is in contrast with “active” microrheology, in which probe particles are externally forced, such as with laser tweezers or magnetic tweezers, and their motions in response to the applied force are tracked [43]–[45]. Additionally, in order to have the ability to control the microenvironment, it is advantageous to perform microrheology in a microfluidic device with easily and precisely tunable inputs, such as interstitial flow and co-culture conditions.

In this study, we demonstrate an integrated approach that applies mitochondria-tracking microrheology in a compartmentalized microfluidic device. Tracking the fluctuations of intracellular organelles has traditionally been performed in 2D. Our approach provides flexibility and practicability for studies analyzing the effects of environmental factors, especially in 3D, on intracellular mechanics. We describe the key steps to enable this to be practiced, and we look into important practical considerations, specifically temperature effects and a comparison between ballistically injected nanoparticle tracking and mitochondria tracking microrheology. We then focus on a key environmental factor that modulates cell behavior – dimensionality – and demonstrate its impact on intracellular mechanics and drug-induced effects, specifically cytoskeletal disruption via Cytochalasin D.

Notably, we primarily use mitochondria as the tracer particles of interest because they are endogenous and exist in high abundance throughout the cell, enabling a spatial distribution of intracellular mechanical properties to be computed for each cell. This is advantageous over ballistic particle injection microrheology, since particle injection efficiency may be low for some cell types thus resulting in only few traceable particles per cell. The number of traceable particles per cell is reduced further over longer term cultures as cells divide. Additionally, it is unclear if the ballistic injection protocol has adverse or transformative effects on targeted cells, as cells are transiently placed under stressful conditions, vacuumed and pressurized, without media. Finally, mitochondria are important multifunctional organelles that play critical roles in cell energetics, behavior, and apoptosis [46]. Thus, the very fluctuations of these tracers could provide direct insights toward mitochondrial transport and cell bioenergetics [47], [48].

## Methods

### Cell culture

MDA-MB-231 metastatic breast adenocarcinoma cells were cultured in DMEM (Dulbecco's Modified Eagle Medium, Life Technologies) supplemented with 10% fetal bovine serum and 1% penicillin-streptomycin at 37°C and 5% CO_2_. These cells expressed GFP-actin via Lifeact-GFP (obtained from Dr. Keiko Kawauchi's lab at the Mechanobiology Institute (MBI), Singapore).

### Integrating microrheology into a microfluidic platform

**Figure 1:**
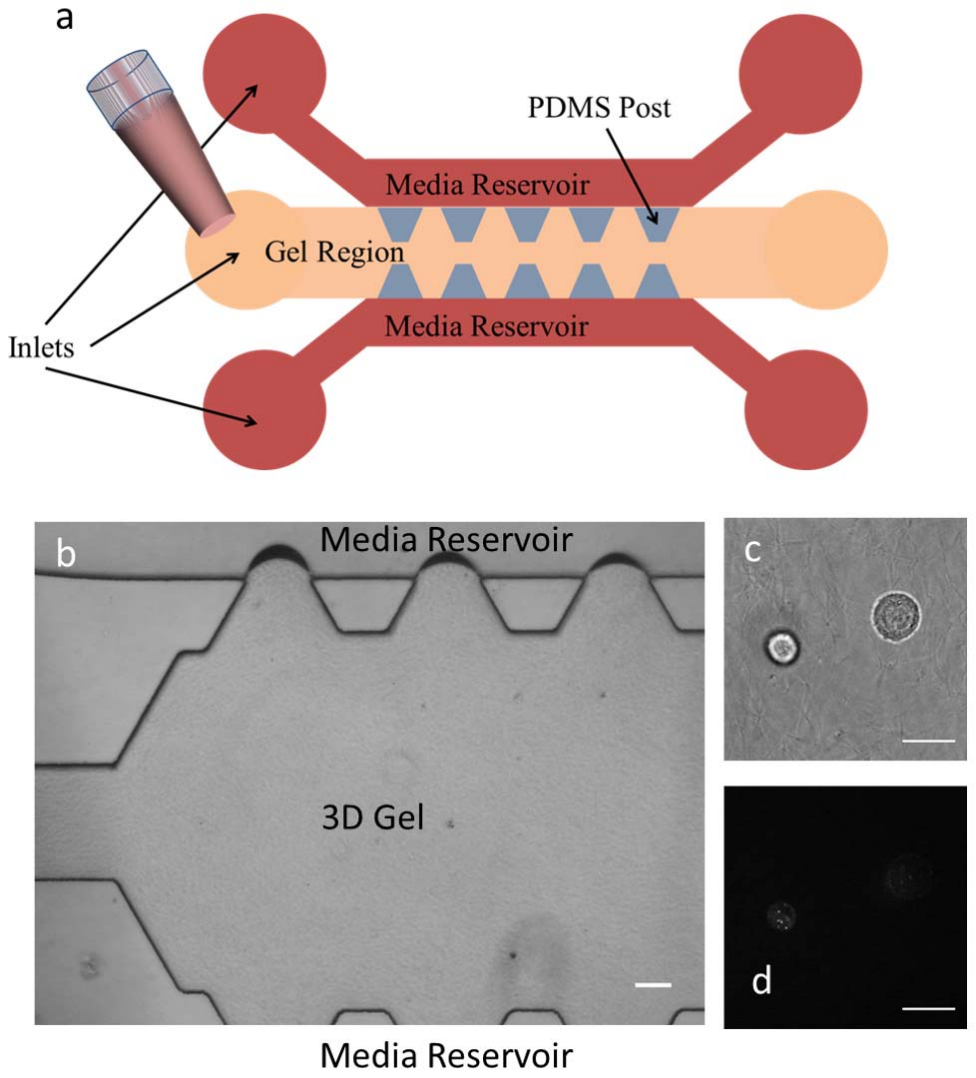
Schematic of the microfluidic device for 3D microrheology experiments. a) Cells in gel are loaded into the gel region via pipetting. The PDMS posts and surface tension keep the gel localized in the gel region. Media is then added to the media reservoirs via pipetting and allowed to diffuse into the gel region. b) Image of the device with collagen gel loaded into and kept contained in the gel region. The device channel height is 200 µm and the inter-post separation is 330 µm. (c) Bright field and (d) fluorescence images of cells embedded in 3D inside the device with ballistically injected 500 nm-diameter fluorescent nanoparticles. The scale bars are 100 µm in (b) and 20 µm in (c) and (d).

The microfluidic device platform used in these experiments is shown in Figs. 1 a and b. An SU8 master of the device can be microfabricated as described previously [49], [50], and PDMS soft lithography can be performed to generate microfluidic devices. The channels are treated with PDL (poly-D-lysine hydrobromide; 1 mg/mL, Sigma-Aldrich) for at least 4 hours and then rinsed twice with sterile water prior to experiments to promote collagen matrix adhesion [49], [50].

To seed the device (Figs. 1a-d), MDA-MB-231 cells are resuspended into 50 µL of complete growth media. 200 µL of type I rat tail collagen solution (Becton Dickinson) (at a final collagen concentration of 2.5 mg/mL and a pH of about 7.4) with about 5 million MDA-MB-231 cells per mL is prepared on ice. A 200 µL pipettor is used to directly inject about 60 µL of this solution into the central channel of the device. Surface tension and microposts help keep the gel solution localized in the central channel (Fig. 1b). After loading, the device with the loaded cells and gel is placed inside a 37°C incubator for 30 minutes to allow for crosslinking and gelation of the collagen. The device is flipped upside down after 1 minute of incubation, followed by 3 more flips 3–5 minutes apart in order to prevent cell sedimentation to the top or bottom surfaces before the collagen fully gels. After the collagen is fully gelled, complete growth media is pipetted directly into the two side reservoirs and the device is incubated for at least 1 day before experimentation.

2D experiments are performed in tissue culture glass-bottom (for high resolution imaging) well-plates without any additional substrate coating. Cells are seeded and cultured in serum containing growth media. MDA-MB-231 cells have been shown to proliferate well on substrates including glass and PDMS in the presence of serum without any other substrate coating [51], as serum contains adhesion proteins [52].

### Microrheology tracer preparation and experiments

Fifteen minutes prior to experimentation, the media at the reservoir regions is replaced with new complete growth media with 500 nM added Mitotracker Red solution (Life Technologies). In about 15 minutes, the mitochondria of cells in the device are fluorescently labeled and traceable. The device is placed in an environmental chamber set to ideal culture conditions (37°C, 5% CO_2_). Cells near the middle of the gel are located and fluorescent images are acquired at high temporal resolution (50 ms per frame) for 100 s. IMARIS (Bitplane, St. Paul, MN) or other auto-tracking software is used to trace the displacement vs. time curve for each tracer and the mean-squared displacements (MSDs) are calculated. Net displacements are subtracted for each tracer to account for drift and persistent motions. All experiments performed in this study were done in an environmental chamber set to 37°C and 5% CO_2_ unless otherwise specified. At least 9 individual cells are studied in mitochondria tracking experiments in each case for 2D and 3D cultures with and without Cytochalasin treatment over multiple plates or devices, with at least 68 mitochondria tracked in each case.

Once the MSDs are computed, post-processing following previous studies to fit MSDs to a locally weighted polynomial and applying an algebraic approximation of the Generalized Stokes-Einstein Relation (GSER) can be performed to compute the shear modulus [20], [41], [42], [53]–[56]:
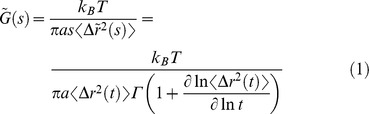
where 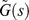 is the shear modulus, *s* is the Laplace frequency, *k_B_* is the Boltzmann constant, *T* is the temperature, *<Δr^2^(t)>* is the MSD (in 3D assuming an isotropic medium), *t = 1/s* is the time interval, *a* is the radius of the tracer particle, and *Γ* is the gamma function. *s* can be substituted by *iω* to obtain the complex shear modulus G^*^(ω) = G′+iG″, where *G′* and *G″* are the elastic and loss moduli, respectively. Note that due to active, non-thermal, motions inside living cells, the GSER becomes unreliable at low frequencies (below 10 Hz) [21], [45], [57], which we consider and discuss in Results.

To use nanoparticles as tracers, a ballistic injection step [58], [59] must be performed prior to cell loading into devices. Briefly, a ballistic particle delivery system (Bio-Rad Laboratories, Carlsbad, CA) is used to propel fluorescent 500 nm carboxylated polystyrene particles into near-confluent cells on a 10 cm^2^ dish.

### Microscopy and image acquisition

A spinning disk confocal microscope was used with dual lasers for exciting GFP and mCherry. Images were acquired using a 63×1.4NA oil immersion objective and an electron multiplying CCD camera (Hamamatsu Photonics, Hamamatsu, Japan). The image plane is focused inside the cell both in 2D and 3D. In 3D, cells in the middle of the gel were chosen to avoid boundary effects. Over the course of image acquisition, which was only 100 s per video, no significant focal drifts were observed. Baseline MSD due to system noise of beads stuck on the surface of a cover glass has been measured to be ∼10^−4^–10^−3^ µm^2^ (N = 30), which is below our average MSDs for mitochondria motion in our results. This baseline measurement is shown in Fig. S1 in Supporting Information S1.

### Cytoskeletal disruption experiments

Cytochalasin D (Sigma-Aldrich) disrupts the cytoskeletal network by capping actin filaments [60]. Cells are treated with 5 µM Cytochalasin D, dissolved in dimethylsulfoxide (DMSO) and mixed in complete growth media, for at least 1 hour prior to the experiments on cytoskeletal disruption.

### Statistical analysis

Error bars are standard error of the mean (s.e.m.) and statistical significance is computed from One-way ANOVA tests for p<0.05 unless stated otherwise.

### Brownian dynamics simulations

Simulations are performed as previously described [61]–[63]. Briefly, a 3D domain is generated with fixed or periodic boundary conditions. A fixed boundary is hard, such that particles cannot penetrate through it. A periodic boundary is one where a particle crossing it comes out on the opposing boundary. Actin monomers are polymerized into filaments in the presence of actin binding proteins (motors and cross-linkers). Motors have two arms, each binding to a different filament, and walk towards the barbed end of the filaments, generating tension, and have mechanochemical rates in accordance with the literature [64]. Cross-linkers are mechano-sensitive and have binding and unbinding rates that respond to force. Simulations are run for several hundred seconds and the system is allowed to reach steady-state.

## Results and Discussion

### Anisotropic local fluctuations and spatial distributions

**Figure 2:**
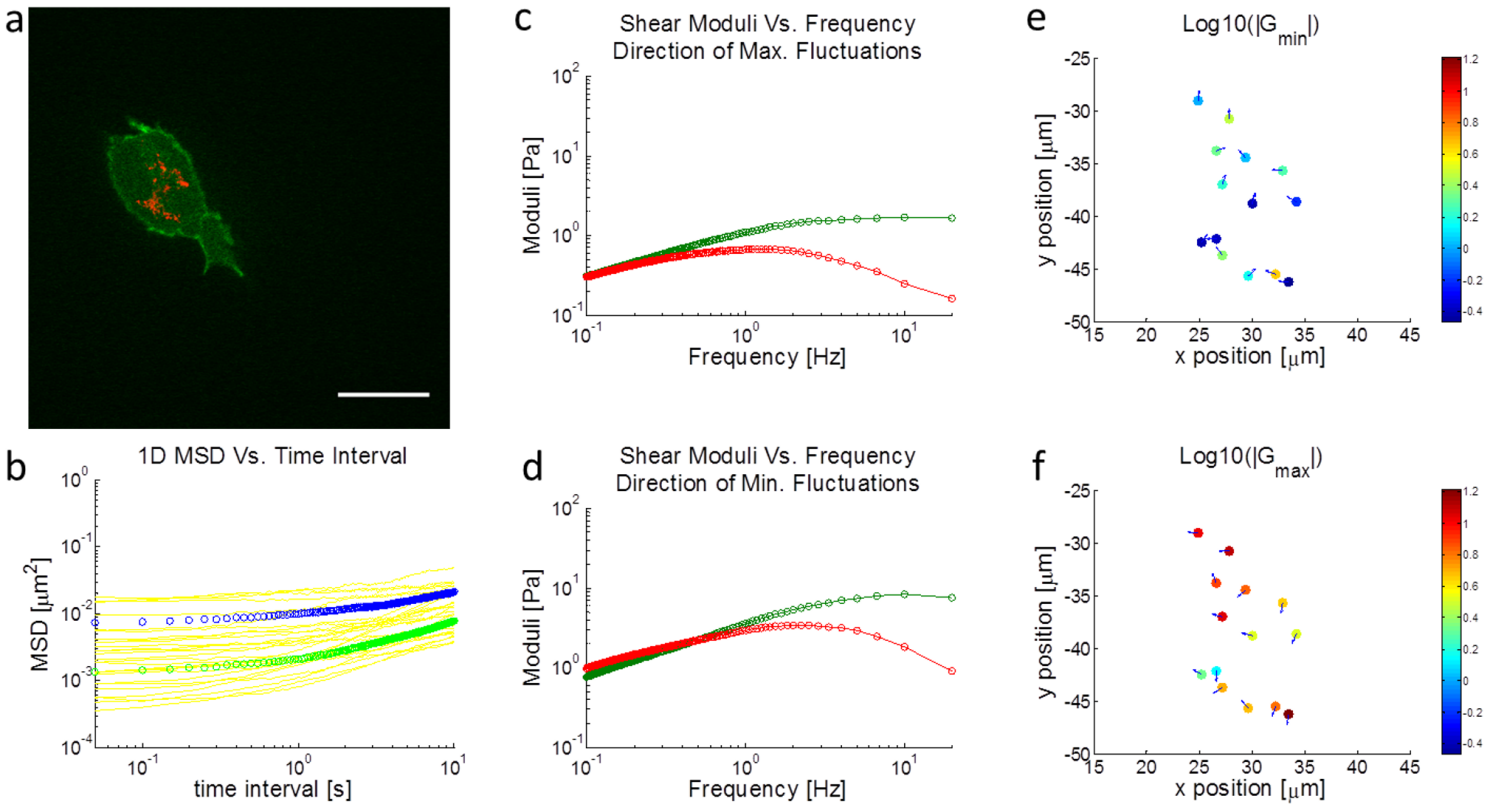
Intracellular microrheology of a single cell in 3D in a microfluidic device. a) Fluorescence image of an MDA-MB-231 cell in 2.5 mg/mL collagen. Actin is green and mitochondria are red. b) 1D MSDs in the two orthogonal directions with the maximum ratio in displacement fluctuations. Blue is the average of the MSDs in the directions of maximum fluctuation and green is the average of the MSDs in the directions of minimum fluctuation. Maximum and minimum directions are determined on a particle by particle basis. Yellow curves represent the 1D MSDs of each individual mitochondrion tracked. c,d) The complex shear modulus G in the directions with maximum (c) and minimum (d) fluctuations. Green is the elastic modulus and red is the loss modulus. e,f) Spatial plots of the log of the magnitude of the shear modulus G in the direction of (e) maximum and (f) minimum fluctuations at 10 Hz. Arrows point in the directions with the maximum (e) or minimum (f) fluctuations. Colors represent the magnitudes of log|G|. This experiment was performed at ambient conditions. The scale bar is 20 µm.

We tracked the fluctuations of fluorescently labeled mitochondria in highly metastatic breast adenocarcinoma cells (MDA-MB-231) in the 2D imaging plane (Fig. 2a) and showed that on average there is local anisotropic motion (Fig. 2b). This motion is likely due to locally anisotropic mechanical properties within the cell [27], although probe asymmetries could also lead to this effect. The anisotropy is also apparent in nanoparticle tracking data (Fig. S2 in Supporting Information S1), suggesting that there is likely anisotropic mechanical properties in the cytoskeleton. To maximize the appreciation of this effect, we rotated the coordinate axes for each probe in each cell such that the variance of the displacements in the two principal orthogonal directions have maximal ratio. As shown in Fig. 2b, the average anisotropy appears to persist for over 10 s, as the 1D MSDs in the two principal orthogonal directions do not overlap. It can also been seen in Fig. 2b that there is substantial heterogeneity in the MSD data of even a single cell. The distribution and average for a single cell, however, does appear comparable to the distribution and average of the bulk data (of 9 cells) shown in Fig. S2a in Supporting Information S1, which suggests that intracellular heterogeneity is a key factor driving heterogeneity in the total data. Thus, considering individual mitochondria fluctuations is important in capturing the heterogeneity in the system.

We then applied the Generalized Stokes-Einstein Relation (GSER) to compute the shear moduli based on 1D displacement data along the principal directions, as shown in Figs. 2 c and d. The average diameter of the mitochondria was calculated to be 140 nm by assuming that particle MSDs scale as the inverse of particle diameter [65], and comparing with average MSDs from 500 nm nanoparticle tracking data (Fig. S2 in Supporting Information S1). Because of the abundance of mitochondria in each cell, spatial distributions of the shear modulus can be mapped, as shown in Figs. 2 e and f (at 10 Hz). The directions of maximum and minimum fluctuations are superposed at each position indicating the local principal directions of anisotropy. Globally, because there were no imposed external forces, there does not appear to be any correlated anisotropy in this cell, as shown in Fig. S3 in Supporting Information S1. At higher frequencies, the elastic modulus is larger than the loss modulus and the material of the cell behaves more solid-like. At lower frequencies, however, GSER becomes unreliable due to non-thermal effects, primarily active fluctuations induced by molecular motors [21], [45], [57], [66]. It is likely that the magnitude of the shear modulus is underestimated at low frequencies because of active stress fluctuations. We considered this in more detail in the modeling section, and for the remaining experimental results we primarily analyzed properties of the intracellular displacement fluctuations (MSDs) rather than computed shear moduli.

### Impact of dimensionality on internal fluctuations

**Figure 3:**
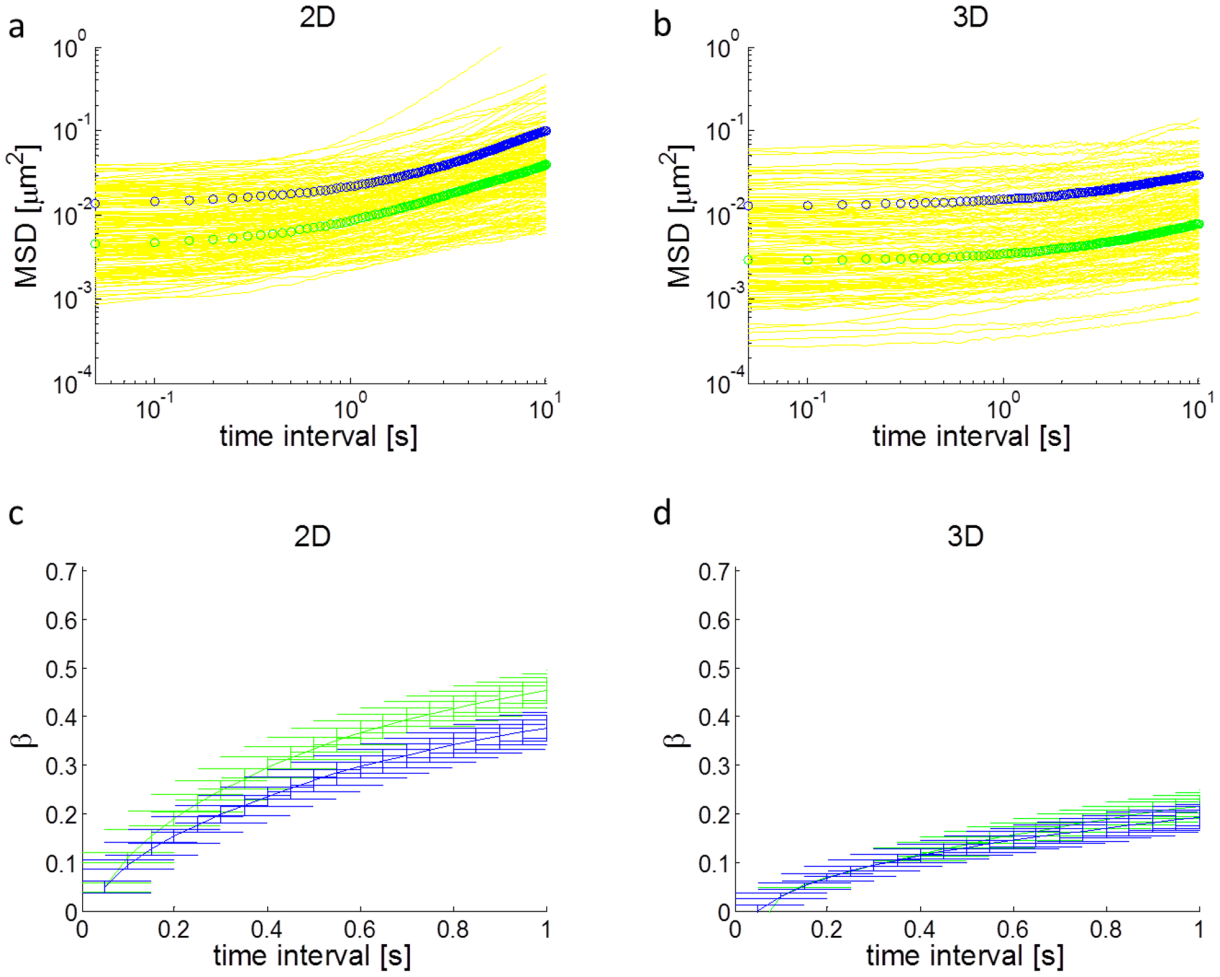
Intracellular fluctuations in 2D vs. 3D. a,b) 1D mitochondria MSDs in the two orthogonal directions with maximum ratio in displacement fluctuations for cells in (a) 2D and (b) 3D. Larger slopes (more fluid-like behavior) and magnitudes are exhibited by cells in 2D at longer time intervals. c,d) Logarithmic time derivative β of MSDs for cells in (c) 2D and (d) 3D in the corresponding 1D directions. Cells in 3D have lower β's, indicating more solid-like behavior. Error bars are s.e.m. The color code of the curves is the same as in Figure 2b.

Next we investigated the impact of dimensionality on mitochondrial fluctuations in the cell. Traditional studies of cell mechanics, including migration and viscoelasticity, are performed on 2D substrates. However, it has emerged from recent studies that cells behave differently when embedded in a 3D microenvironment [67], which is physiologically more relevant. Here we investigated the impact of dimensionality on intracellular mechanical properties. Our results demonstrate that intracellular fluctuations are different in 2D compared to 3D. Specifically, cells in 2D appear to have larger and more fluid-like fluctuations at longer time scales, as shown in Figs. 3 a and b. At a time interval of 10s, the 2D MSD is over three times larger than the 3D MSD.

We then investigated the power-law dependence of the MSDs, as shown in Figs. 3 c and d. The power-law exponent *β* 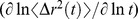 is time dependent and is larger in 2D compared to 3D. At a time interval of 1 s, *β* about doubles in 2D from 3D. A power-law exponent of 0 corresponds to solid-like materials and a power-law exponent of 1 corresponds to fluid-like materials [41], [42]. Thus, 2D cells exhibit more fluid-like internal fluctuations. These results suggest that dimensionality plays an important role in modulating intracellular mechanics. We note here that for 3D experiments, because our microfluidic device is only 200 µm high, cells are always within 100 µm from a rigid boundary (glass or PDMS), which may impact cell behavior. The effects of boundary proximity on intracellular properties will require further studies. In our experiments, we do however select for cells that are surrounded by collagen in all 3 dimensions.

In these experiments, in 2D cells were grown on glass and in 3D cells were embedded in collagen I. The difference in substrate composition could potentially alter cell stiffness by modulating cell adhesion or substrate stiffness. Note, however, the cells used here have been shown to adhere and proliferate well on glass (and other substrates such as PDMS) without additional coating beyond growth media containing serum [51] because of adhesion proteins in serum [52]. Previous studies have shown that increasing substrate stiffness could lead to an increase in cell stiffness [1], [68], [69]. Additionally, inhibiting cell adhesion via integrin blocking may reduce cell stiffness [39], likely by reducing internal prestress. These effects, if present, will induce a vertical shift in the log-log plot of the MSDs, particularly at short time-scales, for which the GSER is valid. In our results, we found that for short times, the magnitudes of the MSDs are comparable between cells in 2D and 3D, suggesting that the stiffness of the cells in 2D and 3D in our experimental conditions are comparable. The difference emerges at long times, where active (motor-induced) motions tend to dominate. This effect has not been previously elucidated based on changing matrix composition alone. These results are also consistent with recent work that demonstrated that inhibiting motor activity via blebbistatin treatment or ATP depletion [45] can alter exclusively the long time characteristics of the MSDs. This suggests that the effect we observed is likely due to a modulation in motor activity, which we further explore in the modeling section.

It is noteworthy that directed active transport, defined as highly persistent (constant velocity) motions that are not random over long times, of mitochondria is likely negligible. These motions would result in MSD = *v^2^t^2^*, where *v* is velocity and *t* is time, so β would equal 2. This does not appear to occur based on recent work [45]. We nevertheless subtracted net displacements from our data, as mentioned in the methods, so our analysis should not depend on directed active transport. However, active fluctuations, meaning persistent but random motions due to motors, are likely not negligible [21], [45], [57]. If active fluctuations are negligible, we expect the log-log plot of the MSDs to appear flat with a weak power-law dependence on time interval (small constant positive slope), consistent with active microrheology methods [43], [45]. Active fluctuations manifest in the long time regime in the MSDs, as shown by the increase in β. This increase is consistent with non-thermally driven motions, as demonstrated in other studies [45], [57] by comparing active and passive microrheology measurements of cytoskeletal networks containing motors.

### Impact of dimensionality on cytoskeletal disruption

**Figure 4:**
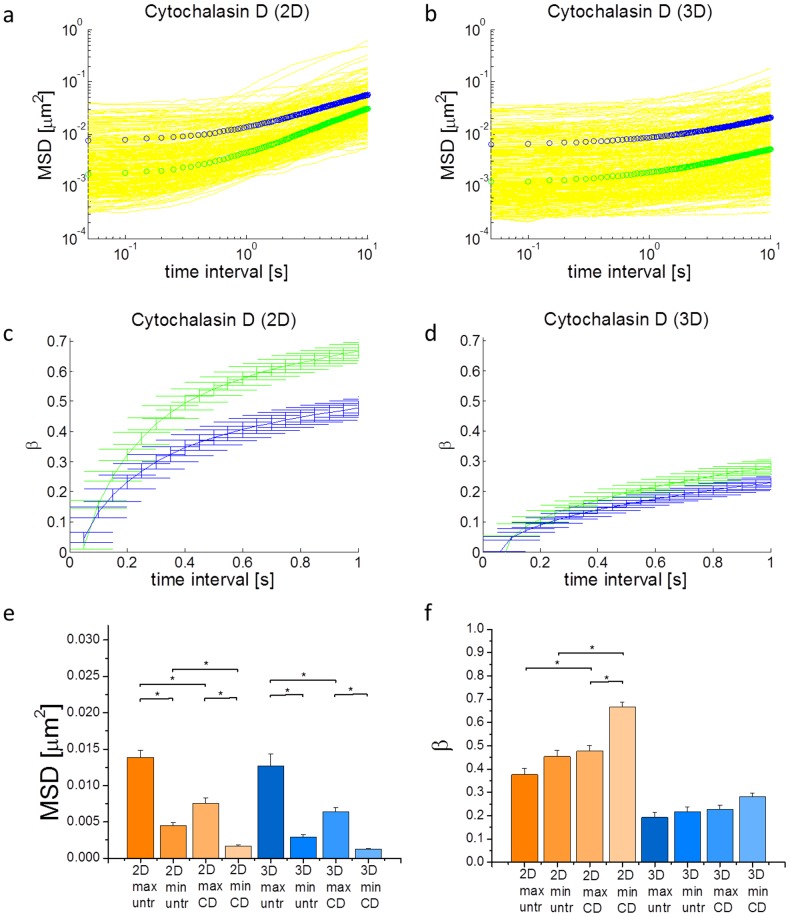
Cytochalasin D treated cells in 2D and 3D. a,b) MSDs of cells treated with Cytochalasin D in (a) 2D and (b) 3D. c,d) β's of cells treated with Cytochalasin D in (c) 2D and (d) 3D. e) Comparison of MSDs at 50 ms of untreated and Cytochalasin D treated cells in 2D and 3D. f) Comparison of β's at 1s of untreated and Cytochalasin D treated cells in 2D and 3D. Max and min indicate the 1D direction of maximum and minimum fluctuations, respectively. “untr” and “CD” indicate untreated and Cytochalasin D treated cells, respectively. Cells in 2D exhibit a more pronounced effect under treatment than cells in 3D. Error bars are s.e.m. * indicates p<0.05. The color code in (a) and (b) is the same as in Figure 2b.

A key goal in understanding cell mechanics in diseases such as cancer is to develop platforms and methods that can screen for therapeutics that modulate cell mechanical properties. In order for effective screening to be performed, however, it is necessary to be reasonably confident that cell responses in the screening assays can reproduce cell responses in physiological environments. Here, our interest is in the effect of the dimensionality of the cellular microenvironment. We have already shown that the mechanical properties of cells, as inferred from MSDs, are different between 2D and 3D environments, which suggests that 2D cell-based assays for cell mechanics may not properly recapitulate 3D physiological cell responses. We next tested whether cytoskeletal disruption would impact cell mechanical properties in different ways in a dimensionality-dependent manner. We disrupted the cytoskeletal actin network with Cytochalasin D, which destabilizes the network by capping actin and inhibiting polymerization [60], and measured intracellular fluctuations in both 2D and 3D (Fig. 4). 5 µM Cytochalasin D treatment for 30 min. has been shown to be sufficient in substantially disrupting intracellular mechanics to a steady level [70], [71]. Our results show that the mechanical response of cells to this treatment appears to be significantly dependent on dimensionality. Cytochalasin D reduces intracellular fluctuations at short time intervals, which suggests an increase in network stiffness, and increases *β* (at least in 2D) at longer time intervals, which suggests an increase in fluidity (Figs. 4 e and f). The net impact of Cytochalasin D appears to be more pronounced at 2D, at least with respect to changes in *β*.

**Figure 5:**
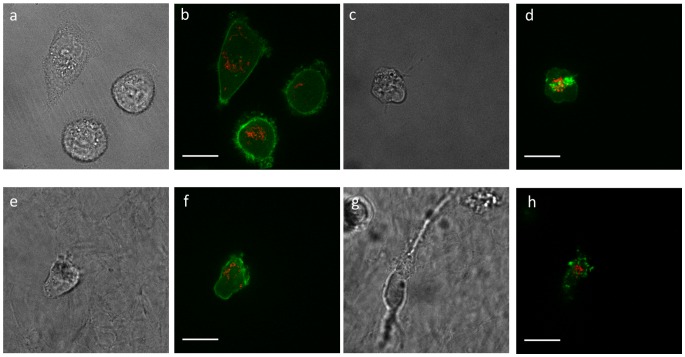
Images of MDA-MB-231 cells in 2D and 3D with and without Cytochalasin D treatment. a–h) Bright field and fluorescence images of (a,b) untreated cells in 2D, (c,d) Cytochalasin D treated cells in 2D, (e,f) untreated cells in 3D, and (g,h) Cytochalasin D treated cells in 3D. Green and red represent actin and mitochondria, respectively. The scale bar is 20 µm.

We then considered the morphological impact of Cytochalasin D treatment in 2D and 3D and showed that in both cases, the cytoskeleton is clearly disrupted, as demonstrated by punctate actin aggregation (Fig. 5). We speculate that a decrease in fluctuations at low time intervals may be due to inclusion of the probes within a region of increased local actin concentration due to actin aggregation. The fluid-like behavior at longer time intervals is likely due to decreased actin network connectivity, since aggregates are formed and the initial homogeneous distribution in the cytosol is collapsed. Additionally, our results show that while the morphological impact of Cytochalasin D is apparent in both 2D and 3D, the measured mechanical effects are more pronounced in 2D.

### Modeling

**Figure 6:**
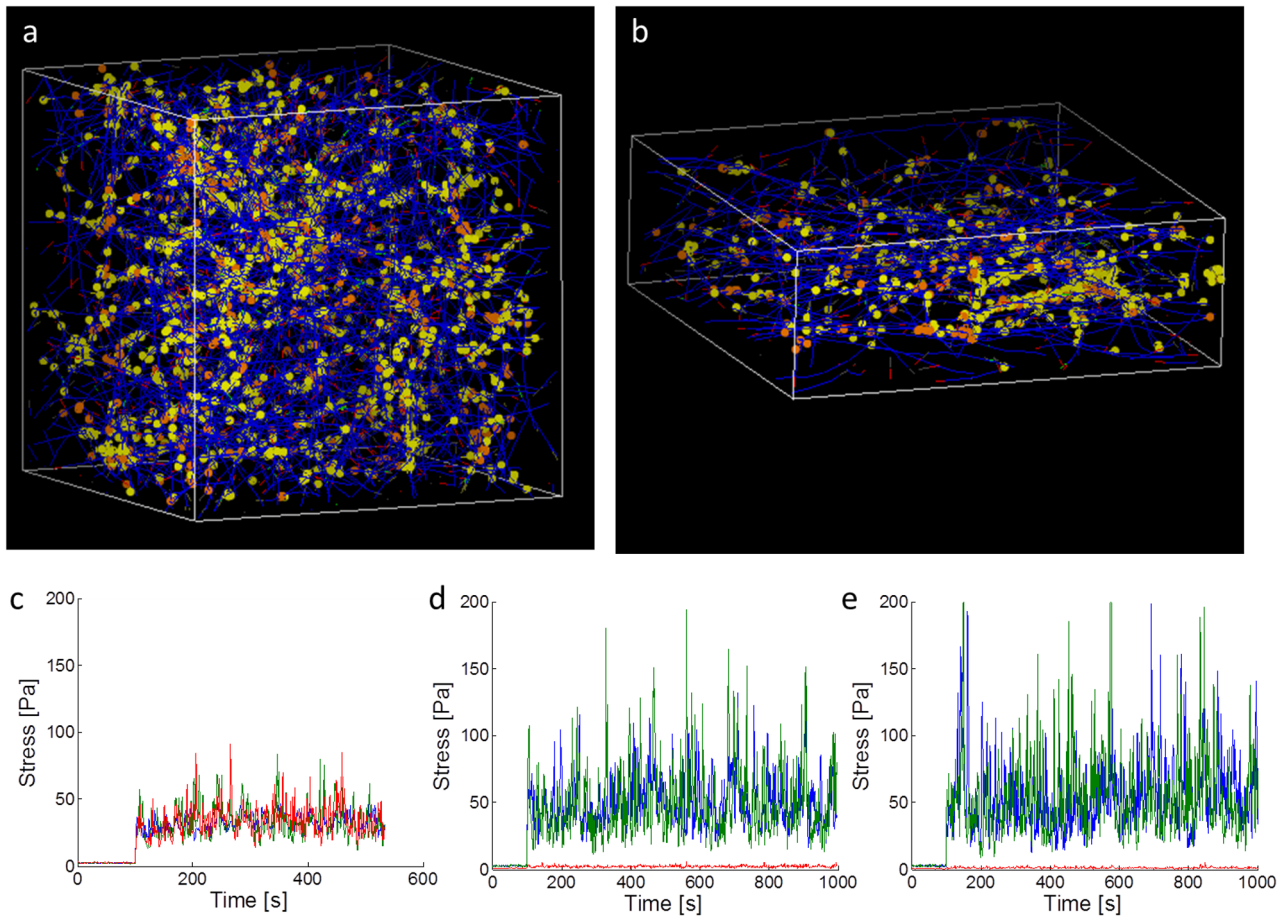
Brownian dynamics simulations of active actin networks. a) A 3D 3×3×3 µm^3^ (in the x, y, and z directions, respectively) domain with periodic boundary conditions on all sides, mimicking the cytoskeletal network in 3D. Actin filaments (blue) are polymerized from G-actin monomers and allowed to bind to myosin II motors (orange) and crosslinkers (yellow). Motors walk along two filaments toward their barbed ends, generating internal stress. b) A 3D 3×3×1 µm^3^ domain with periodic boundary conditions in the x and y directions and fixed boundaries in the z-direction, mimicking a 2D cell configuration. The actin network is more planarized, as the filaments align along the x-y plane. c,d,e) Average internal stresses in the x (blue), y (green), and z (red) directions for (c) a 3D network, (d) a 2D network with 1 µm height in the z-direction, and (e) a 2D network with 500nm height in the z-direction. 2D networks have larger stress fluctuations from the mean in the x and y directions (the plane of interest in experiments), but reduced fluctuations in z, as compared to 3D networks. 3D networks have comparable average internal stresses in all 3 dimensions. 2D networks have reduced average stresses in the z-direction (perpendicular to the plane of filament alignment), generating mechanical anisotropy. The stresses for each time point for each simulation were calculated by summing the tensional forces of filaments crossing a total area of 9 µm^2^ in each direction. Motors start walking and generating tension along filaments at time  = 100 s.

In order to gain insights into the physical mechanisms that may contribute towards dimensional modulation of intracellular behavior, we performed Brownian dynamics simulations of actin networks in 3D and under conditions mimicking “2D”. 3D simulations are conducted in a 3×3×3 µm^3^ cubical domain with periodic boundary conditions. 2D simulations are conducted in a 3×3×1 µm^3^ domain with periodic boundaries in the x and y directions but with fixed boundaries in the 1 µm-thick z-direction. Super-resolution imaging studies have shown that the height of the cytoskeleton in 2D is submicron [72]. Typical confocal images of MDA-MB-231 cells in 2D and 3D are shown in Fig. S4 in Supporting Information S1, which elucidates dimensional effects on cytoskeletal morphology. Our simulation results demonstrate that 2D networks cause the actin filaments to align in the x-y plane and the stress fluctuations (from mean stress) in those directions increase; this coincides with a decrease in stress fluctuations in the z-direction, as shown in Fig. 6. The stress fluctuation distributions are shown in Fig. S5 in Supporting Information S1, illustrating that the variance of the data over time is larger in the x-y plane in 2D.

In our simulations, we specifically considered stress fluctuations because they are the source of motions in the cytoskeleton. When motors are inactive, as in the first 100 s of the simulations shown in Figs. 6 c-e, stress fluctuations are low, since thermal collisions have extremely low persistence. When motors are active, t>100 s, the average stress level is increased, due to motors walking along filaments and generating tension (prestress) in the network. Additionally, the stress fluctuates over time as tension is generated and released due to motor walking and crosslinker binding and unbinding, causing internal movements. Displacement fluctuations (MSDs) are proportional to the ratio of the internal force fluctuations to the shear modulus of the material, in accordance with GSER (for the passive case with inactive motors) and modifications to the GSER that incorporate active sources of internal force [21], [73]. We also derive a model that relates internal energetics to internal motions later. What we essentially show with our simulation results is that the magnitude of the force fluctuations is different in 2D compared to 3D when all other parameters are the same, *i.e.* concentration, mechanics, and kinetics of actin, motors, and crosslinkers. Knowing that there is a difference in the source (force) that drives internal motions now allows us to explore the implications of this property on the behavior and qualitative features of intracellular movements.

Therefore, we next aimed to quantitatively describe how alterations in active internal stress fluctuations, such as from dimensional modulation, can lead to distinct changes in the characteristics of MSDs of intracellular particles. We developed a simple effective temperature model, which we derived in Text S1 in Supporting Information S1. In this model, non-thermal, motor-induced active stress fluctuations manifest in the following form in the Laplace frequency domain:
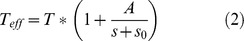
where *T_eff_* is the effective temperature including non-thermal effects, *T* is the actual temperature, *A* corresponds to the amount of motor activity per unit time that leads to active stress fluctuations (and we show in Text S1 in Supporting Information S1 that *A* is proportional to the added kinetic energy in the system induced by motors), *s* is the Laplace frequency, and *s_0_* is a characteristic frequency below which the effect of motors simply leads to a constant, plateaued scaling of the actual temperature. Essentially, this model states that at relatively high frequencies (s>>s_0_), there is an extra frequency dependent term in the GSER. At relatively low frequencies (s<<s_0_), the impact of motor activity approaches a plateau. This form factor is consistent with trends observed in previous experimental studies, which also suggest that the experimentally relevant regime is s>>s_0_ [21], [66]. For systems that exhibit larger stress fluctuations, *A* is larger. Using this model, the GSER then becomes:
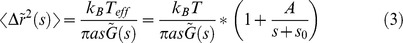
and we can now consider two limiting cases: elastic solids and viscous fluids. For a solid (or more precisely in this case harmonically bound Brownian particles [74]–[76], 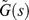 is constant, now denoted as *G*, and the GSER states:
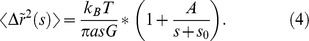
In the limit that s>>s_0_, the Laplace transform pair of the MSD in the time domain is:
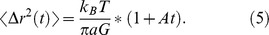
Similarly for a purely viscous Newtonian fluid, 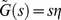, where η is the viscosity, a constant. In this case: 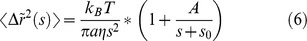
which, again for s>>s_0_, in the time domain is:
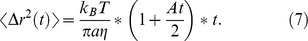
We speculate that for a power-law viscoelastic material, the Laplace transform pair of the MSD for s>>s_0_ may take the forms:
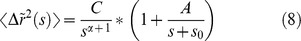





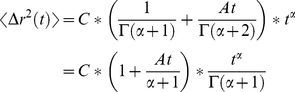
(9)where *C* and α (between 0 and 1) are constants and *Γ* is the gamma function.

**Figure 7:**
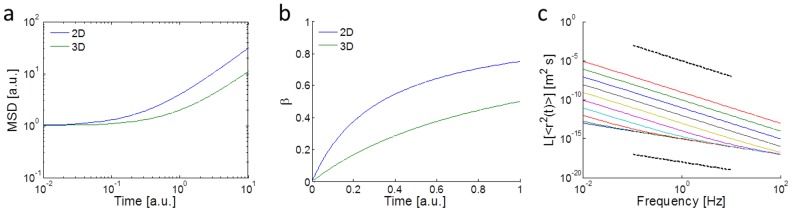
Simulated MSDs and β's for 2D and 3D systems using the effective temperature model to account for active stress fluctuations. a,b) Simulated (a) MSDs and (b) corresponding β's for cells in 2D (blue) and 3D (green). Cells in 2D have a larger stress fluctuation term *A* (3 times larger in this case). c) The Laplace transform of the MSDs based on the effective temperature model for different *A*'s (from 0.001 to 10^6^ [1/s]) and fixed *s_0_* = 0.001 Hz. The simulations in (c) assume an elastic material (G is constant and equal to 20 Pa) with 70 nm-radius tracer particles at T = 300 K and power-law trends are attributed to the effective temperature. The bottom and top dotted lines have slopes of 1 and 2, indicating thermal and super-thermal (with motor activity) spectral trends, respectively.

These relations predict the two regimes seen in our experimental data for MSD's and the trend for β's and suggest an explanation for the impact of dimensionality on intracellular mechanics and dynamics. The planarization of the cytoskeletal network in 2D leads to an enhancement in motor-induced stress fluctuations in that plane and thus an increase in the factor *A* in the equations. In the time domain, an increase in *A* leads to an earlier onset of the second power-law regime and a corresponding increase in β during those time intervals. The predicted trends from this model for 2D (larger *A*) and 3D (smaller *A*) are shown in Fig. 7 and are in agreement with the experimental data shown in Fig. 3. Fig. 7c shows the simulated frequency spectrum of the MSD based on the effective temperature model for different levels of motor activity. These trends are comparable to previously observed experimental microrheology studies for various cell types [21], [66].

In addition to giving mechanistic insights towards the impact of dimensionality on intracellular motions, the Brownian dynamics simulation results shown in Fig. 6 also reveal potential mechanisms driving local mechanical anisotropy in cytoskeletal networks. Anisotropy in viscoelasticity may arise from anisotropy in the alignment of filaments in the cytoskeleton. This is observed in the “2D” simulations. Due to alignment of filaments in the x-y plane in the 2D simulations, the magnitude of the average motor-induced stress is much lower in the z-direction, as shown in Figs. 6 d and e. The amount of internal stress is proportional to the stiffness of the cell, as demonstrated by prior work [77], [78] measuring both cell traction and rheological properties. Thus, our simulations suggest that anisotropy could arise due to filament alignment and that the direction perpendicular to the plane of filament alignment will exhibit reduced stress. The shear modulus in the direction of alignment should be decreased, assuming that the Young's and shear moduli are proportional and according to the tensegrity model [77]. This is consistent with previous experimental results that demonstrated that intracellular MSDs are larger along the direction of induced alignment of endothelial cells under shear flow [27]. Taken together, our simulation and experimental results suggest that there may be local intracellular network alignment leading to local anisotropy in mechanical properties.

### Effects of ambient conditions and tracer type

We tested for potential differences in mechanical properties of cells in 3D inside an incubation chamber (37°C, 5% CO_2_) and at ambient conditions. Our results show that the trends and magnitudes of the MSDs are comparable (Figs. S2 a and b).

Additionally, we performed microrheology experiments in the microfluidic device in 3D using ballistically injected nanoparticles (500 nm diameter). The motion of intracellular particles should scale as the inverse of their diameters for particles larger than the mesh size of the cytoskeleton, which is around 50 nm [45], [79], [80]. The trends of the MSDs are comparable between mitochondria-tracking and particle-tracking microrheology (Fig. S2 in Supporting Information S1), suggesting that mitochondria are effective probes for measuring intracellular mechanics. Differences are likely attributed to probe geometries or alterations in cell mechanics due to bead injection. There may also be mitochondria-specific motors that induce further distinctions and enhance mitochondria fluctuations, although the overall features of the motions of endogenous and ballistically-injected exogenous particles are similar [45]. Mitochondria and other intracellular granules have been previously used in a number of 2D cell microrheology studies [21], [23], [27].

### Conclusions

Based on our simulations and experimental results, dimensionality and cytoskeletal disruption both have distinct effects on intracellular mechanics. The more sold-like motion of intracellular particles in 3D (lower β) in Fig. 3 is likely due to the geometry of the cytoskeleton. In 2D, cells and their cytoskeleton are more planar [72], whereas in 3D, the cytosolic cytoskeletal network is more isotropic since there is less geometric constraint. Based on our simulations, the result of this geometric difference is that motor activity is enhanced in the imaging (x-y) plane in 2D but not in 3D. Enhanced motor activity leads to increased stress fluctuations (from mean stress) in the cell, giving rise to increased active internal motions (MSDs at long time scales). Therefore, based on the effective temperature model, the enhancement in stress fluctuations in 2D leads to an earlier emergence of the second power-law (larger β) regime in the MSDs.

Disrupting the cytoskeleton via Cytochalasin D demonstrated visually the aggregation of actin (Fig. 5), with localized regions of increased actin concentration. Increased local concentration around the probes will lead to increased local stiffness, which would suppress the MSDs. This is consistent with our experimental results at short time intervals (Fig. 4). For longer time intervals (1 s), there is an increase in β for Cytochalasin D treated cells. This may be due to a loss of connectivity in the cytoskeleton due to actin aggregation, such that the network inside the cell is no longer well percolated. This loss of percolation leads to a decrease in global stress inside the cell. This is consistent with previous experiments demonstrating that Cytochalasin D treatment abolishes cell traction forces [71]. A decrease in internal tension and network connectivity leads to networks that have decreased global stiffness and more fluid-like behavior [63], [77], [78], [81]–[84], since the network becomes more like disconnected aggregates diffusing in the cytoplasmic fluid.

While dimensionality is a fundamental feature of all physical systems, its impact on cell behavior is not well understood. This is especially important to consider, since many cell biology and mechanics experiments have been performed in 2D environments, which is not physiologically accurate. Towards that end, we have demonstrated the ability to integrate 3D cell culture in an environmentally tunable microfluidic platform with intracellular particle tracking microrheology, thus illustrating and enabling a practical means to study intracellular mechanics on-chip in 3D. Key advantages include lower volumes of reagents required and a more tunable microenvironment. We then demonstrated the importance of dimensionality in altering intracellular mechanics and when testing for effects in response to cytoskeletal disruption. We found that cells in 3D exhibit mechanical characteristics distinct from 2D, and their mechanical response to cytoskeletal disrupting drugs are different from cells in 2D. Cells in 2D appear to have more fluid-like intracellular fluctuations and their response to Cytochalasin D treatment is more pronounced than cells in 3D. Finally, through Brownian dynamics simulations of active actin networks and an effective temperature model, we showed that dimensionality can impact the magnitude of the non-thermal motor-induced stress fluctuations inside the cell, leading to differences in intracellular dynamics and particle motion. Cytoskeletal remodeling due to actin disrupting drugs such as Cytochalasin D is likely impacted by motor activity which is prominent throughout the cell, and enhanced activity in 2D may be responsible for the more pronounced effects seen in our experiments. Future studies exploring the relation between polymerization dynamics, which are altered by Cytochalasin and intracellular actin regulatory proteins such as Mena [85], and cytoskeletal tension and morphology in the presence of motor activity, can provide new insights toward the fundamental mechanical state of individual cells as well as disease states in cancer cells.

Furthermore, during the metastatic process, cancer cells must undergo many instances in which dimensional modulation plays a role. For instance, as individual cells invade through small pores of the tumor ECM (especially non-proteolytically), intra- and extravasate across tight endothelial layers, and obstruct small microvessels during circulation, they exhibit substantial deformations that can significantly squeeze even the nucleus [34], [51], [86], [87]. These events, which induce geometric confinement, can effectively cause the cell to behave mechanically, at least locally, as though it were constrained in a 2D or even 1D environment, thus altering intracellular fluctuations, transport, and motor behavior.

The intracellular space is also crowded and compartmentalized, and diffusion of important molecules such as adenosine phosphates (ATP and ADP) is slow (compared to free diffusion in water) [88]–[90]. Motor activity and active fluctuations may play an important role in transporting sources of ATP by actively redistributing mitochondria in a fluid-like manner over long time scales. Our results show that dimensional modulation from 3D to 2D shortens the time interval before the onset of fluid-like mitochondrial motions, which may alter ATP-dependent and bioenergetic processes as well as the transport of macromolecules in the cell.

Dimensionality, a prominent feature in the biological landscape, has characteristic effects on intracellular mechanics, with implications on internal force generation, transport, and mitochondrial dispersion. Properly recreating the dimensionality of physiological environments in *in vitro* systems may elicit relevant behavior in pathological processes.

## Supporting Information

Supporting Information S1 Contains all supporting information files, including **Text S1**. Derivation and assumptions of the effective temperature model; **Figure S1**. Baseline noise in our experimental setup; **Figure S2**. Mitochondria and nanobead-tracking microrheology at ambient conditions; **Figure S3**. Directions of alignment along maximum 1D MSDs for cell in Fig. 2; **Figure S4**. 3D confocal images of GFP-actin in MDA-MB-231 cells; **Figure S5**. Stress fluctuation distribution from Brownian dynamics simulations.
(PDF)
