# Supplementary material for: Impact of Dimensionality and Network Disruption on Microrheology of Cancer Cells in 3D Environments
Source: PLoS Comput Biol. 2014 Nov 20;10(11):e1003959. doi: 10.1371/journal.pcbi.1003959 (PMC4238946; doi:10.1371/journal.pcbi.1003959)
Supplement: Supporting Information S1 — Contains all supporting information files, including Text S1. Derivation and assumptions of the effective temperature model; Figure S1. Baseline noise in our experimental setup; Figure S2. Mitochondria and nanobead-tracking microrheology at ambient conditions; Figure S3. Directions of alignment along maximum 1D MSDs for cell in Fig. 2; Figure S4. 3D confocal images of GFP-actin in MDA-MB-231 cells; Figure S5. Stress fluctuation distribution from Brownian dynamics simulations. (PDF) [file pcbi.1003959.s001.pdf]

Supplementary Information

SI Text 1: Derivation and assumptions of the effective temperature model

We assume that the displacements of intracellular particles are due to passive thermal activity and active motor-induced activity and that the active and passive processes are independent. In 1D:

$$x_{total} = x_{thermal} + x_{motors} \qquad (1)$$

where x is the displacement and the subscripts indicate the driver of the displacement. For a random process, we expect the displacements to be random fluctuations with a mean of 0:

$$\langle x_{total} \rangle = \langle x_{thermal} \rangle = \langle x_{motors} \rangle = 0 \qquad (2)$$

where < > denotes ensemble average. Assuming independent random processes for $x_{thermal}$ and x_{motors}, for the mean squared displacements we have:

$$\begin{aligned} \langle x_{total}^2 \rangle &= \langle x_{themal}^2 \rangle + \langle x_{motors}^2 \rangle + 2\langle x_{thermal} x_{motors} \rangle \\ &= \langle x_{themal}^2 \rangle + \langle x_{motors}^2 \rangle. \qquad (3) \end{aligned}$$

Assuming the medium is a Newtonian fluid, we can solve for the mean squared displacements separately for thermal and non-thermal fluctuations using the Langevin equation:

$$m\dot{v} = -bv + F(t) \qquad (4)$$

where v is the velocity, $\dot{v}$ is the time derivative of the velocity, b is the damping factor which is assumed to be constant, m is the mass of the particle being tracked, and $F(t)$ is the time dependent driving force, which is random and uncorrelated over time in this case. The general solution for the mean squared displacement is [1,2]:

$$\langle x^2 \rangle = \frac{4KE}{b}\left(t - \frac{1}{\gamma} + \frac{e^{-\gamma t}}{\gamma} \right) \qquad (5)$$

where $\gamma \equiv b/m$ and KE is the average kinetic energy of the system. For the case of both thermal and motor-induced activities:

$$\langle x_{total}^2 \rangle = \frac{4KE_1}{b_1}\left(t - \frac{1}{\gamma_1} + \frac{e^{-\gamma_1 t}}{\gamma_1} \right) + \frac{4KE_2}{b_2}\left(t - \frac{1}{\gamma_2} + \frac{e^{-\gamma_2 t}}{\gamma_2} \right) \qquad (6)$$

where the subscripts *1* and *2* refer to properties associated with the thermal and motor-induced fluctuations, respectively.

For the thermal case, $b_1 = 6\pi\eta a$, where a is the radius of the fluctuating particle and η is the viscosity, and $KE_1 = k_B T/2$, which is in accordance to the equipartition theorem for 1D translational motion. For the motor-driven fluctuations, we assume that motors have additional persistence such that the damping factor b_2 is different and smaller than b_1. For a 3D isotropic medium the mean squared displacement is 3 times as large and the factor of 3 can be absorbed into the KE term, and we now refer to the mean squared displacement as $<r^2> = <x^2> + <y^2> + <z^2>$. For $t >> 1/\gamma_1$, thermal motion is diffusive:

$$\left\langle r^2(t)\right\rangle = \frac{4KE_1}{b_1}t + \frac{4KE_2}{b_2}\left(t - \frac{1}{\gamma_2} + \frac{e^{-\gamma_2 t}}{\gamma_2}\right)$$
$$= \frac{4KE_1}{b_1}t*\left(1+\frac{\gamma_1 * KE_2}{t*KE_1}\left(\frac{t}{\gamma_2}-\frac{1}{\gamma_2^2}+\frac{e^{-\gamma_2 t}}{\gamma_2^2}\right)\right)$$
$$= t\frac{k_BT}{\pi\eta a}\left(1+\frac{A}{t}\left(\frac{t}{\gamma_2}-\frac{1}{\gamma_2^2}+\frac{e^{-\gamma_2 t}}{\gamma_2^2}\right)\right) \qquad (7)$$

where $A \equiv \gamma_1 KE_2/KE_1$. In the Laplace frequency domain:

$$\left\langle \tilde{r}^2(s)\right\rangle = \frac{k_BT}{\pi\eta a s^2}\left(1+\frac{A}{s+s_0}\right) \qquad (8)$$

where s is the Laplace frequency and $s_0 \equiv \gamma_2$ is the characteristic frequency. $T(1+A/(s+s_0))$ is now interpreted as the effective temperature. For $t << 1/\gamma_2$:

$$\left\langle r^2(t)\right\rangle = t\frac{k_BT}{\pi\eta a}\left(1+\frac{At}{2}\right). \qquad (9)$$

Also note here that the average kinetic energy of the system should be proportional to the average of the square of the stress fluctuations shown in Fig. 6 and SI Fig. 3. The stress here is a measure of the tension on actin filaments in the network over a defined area. The stress fluctuations are essentially due to actin filaments stretching and relaxing (thermally and via motor activity) from their steady-state stretched lengths. If we assume that actin filaments are linear elastic, then $<S^2> \sim <k^2L^2>$, where S is the stress fluctuation from mean stress, k is the

spring constant of actin, and L is the deviation of the length of the filaments from their steady-state lengths. Note that due to motor activity, there is prestress in the network so the average stress is non-zero as shown in Fig. 6. $<L^2> \sim v_0^2\ t_p^2$, where v_0 is the instantaneous speed of the change in length due to the source (thermal or motors) and t_p is the persistence time of the source (thermal or motors) in its direction of motion. t_p of thermal collisions should be much lower than that of motors, and v_0^2 is proportional to kinetic energy. Therefore $<S^2> \sim k^2\ t_p^2\ KE$, so when comparing the stress fluctuations of the same material due to the same source, such as motors, increased $<S^2>$ indicates increased KE, and increased KE_{motors} leads to an increase in A in the effective temperature model.

References

1. Uhlenbeck GE, Ornstein LS (1930) On the Theory of the Brownian Motion. Physical Review 36: 823-841.
2. Li T, Raizen MG (2013) Brownian motion at short time scales. Annalen der Physik 525: 281-295.

SI Figure 1

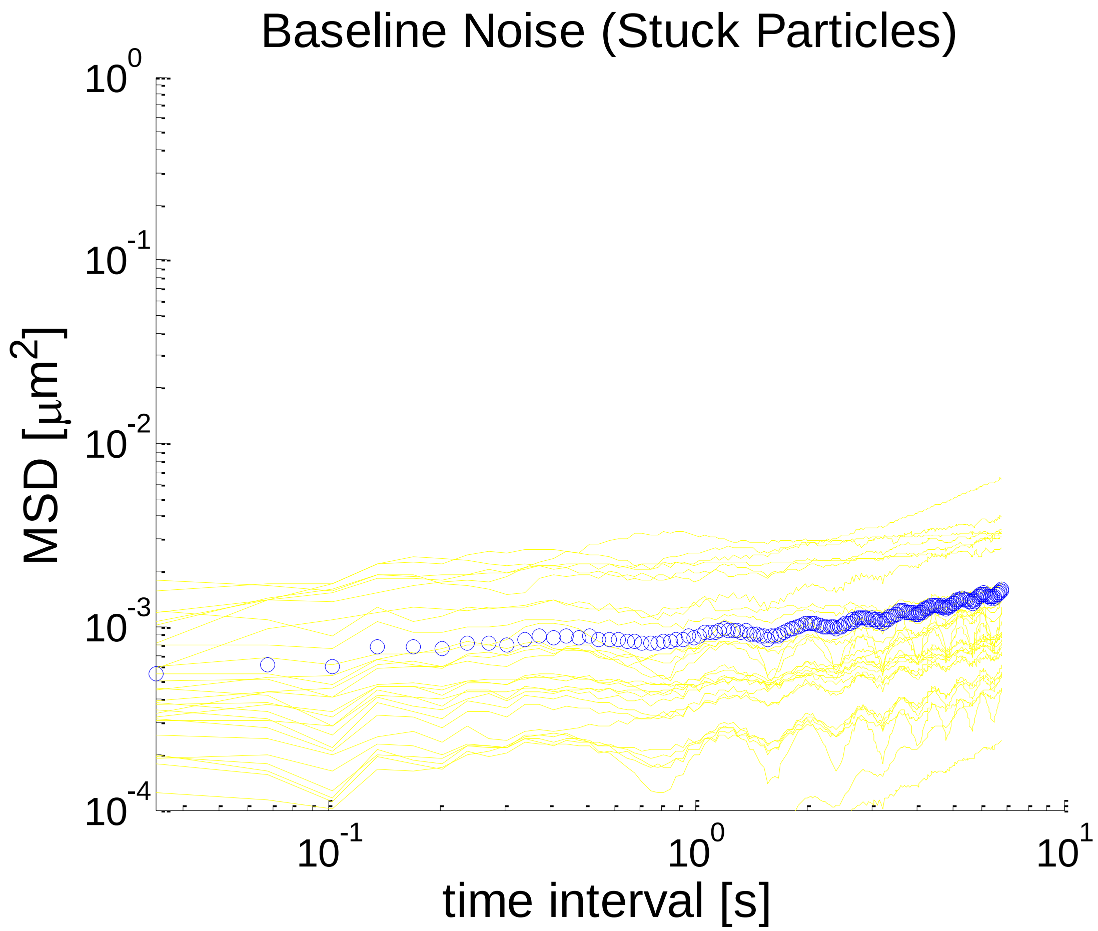

SI Figure 1: Average (blue) and individual (yellow) 2D MSDs of nanoparticles stuck to the surface of a glass slide. This is the baseline noise in our experimental setup.

SI Figure 2

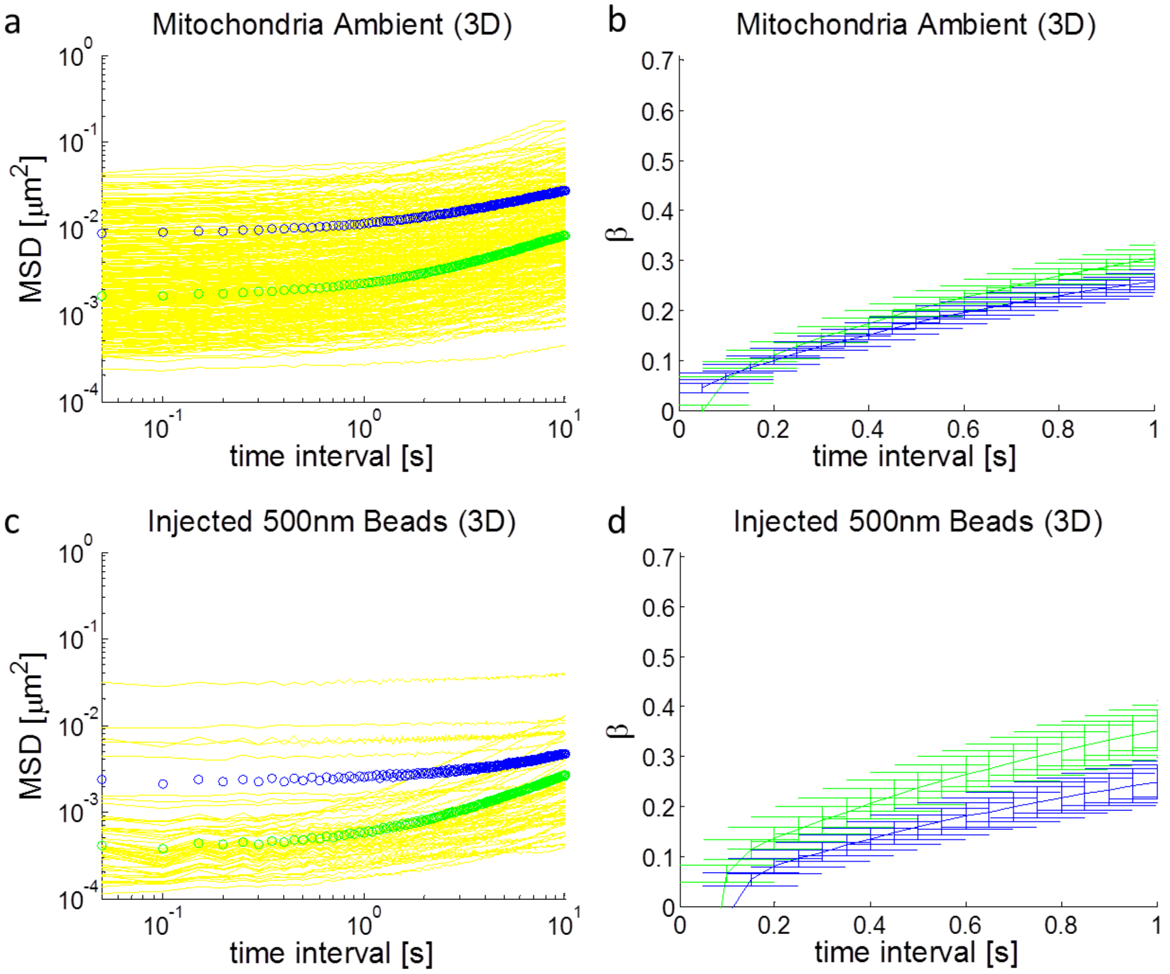

SI Figure 2: Mitochondria and ballistically injected nanobead-tracking microrheology of MDA-MB-231 cells at ambient conditions. Cells were embedded in 3D in collagen in the microfluidic device. a,b) Mitochondria-tracking 1D (a) MSDs and (b) β's in the directions of maximum (blue) and minimum (green) fluctuations. N = 166 mitochondria. c,d) 500nm-diameter polystyrene nanobead-tracking 1D (c) MSDs and (d) β's in the directions of maximum (blue) and minimum (green) fluctuations. N = 30 beads. Experiments here were performed at ambient conditions. Error bars are s.e.m. The color code of the curves is the same as in Figure 2b.

SI Figure 3

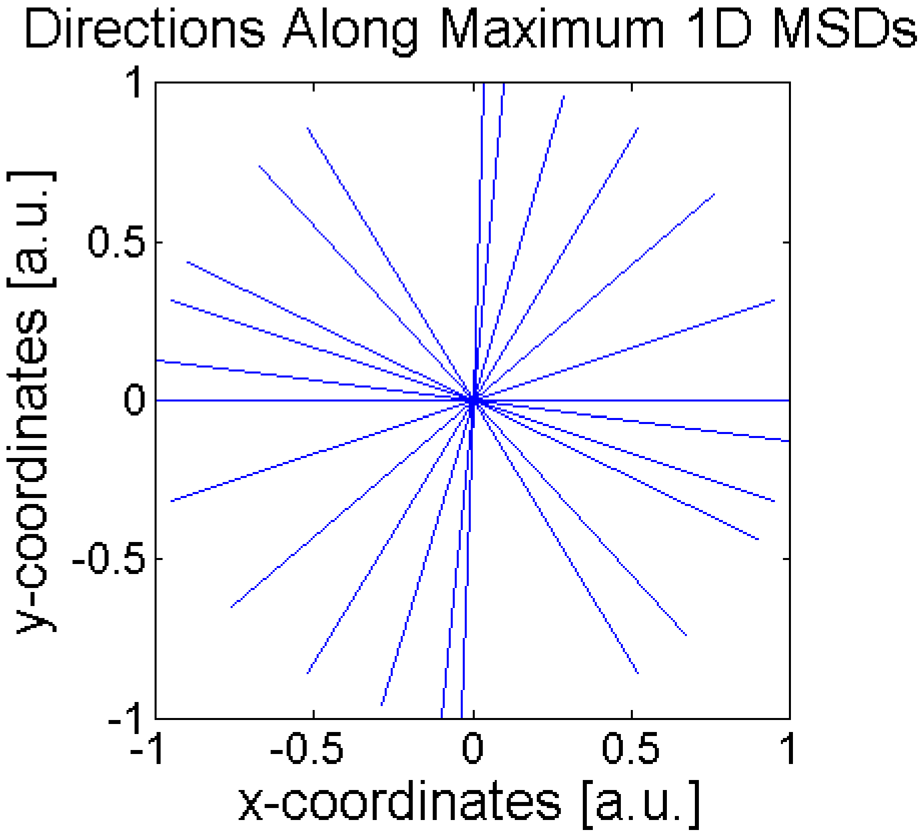

SI Figure 3: Directions of alignment along maximum 1D MSDs for cell in Fig. 2. There does not appear to be global anisotropy in the cell.

SI Figure 4

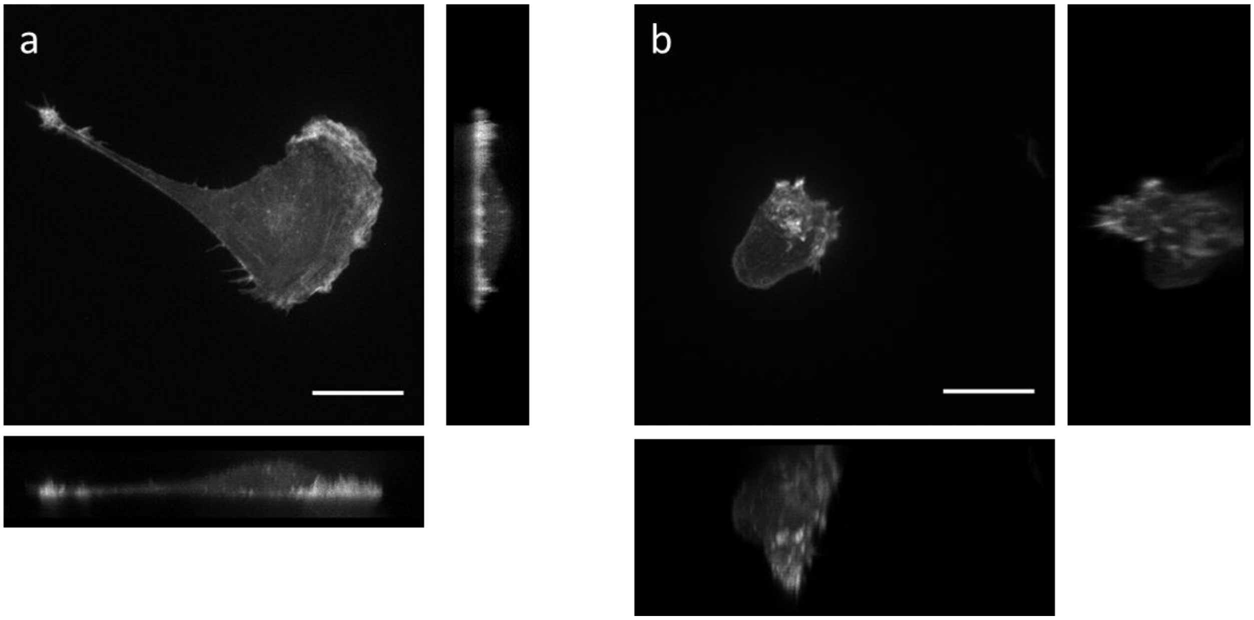

SI Figure 4: 3D confocal brightest point projections of GFP-actin expressed in MDA-MB-231 cells a) on a 2D substrate and b) embedded in a 3D collagen matrix along the xy (center), xz (bottom), and yz planes (right). The scale bar is 20μm and is equal along all 3 image planes.

SI Figure 5

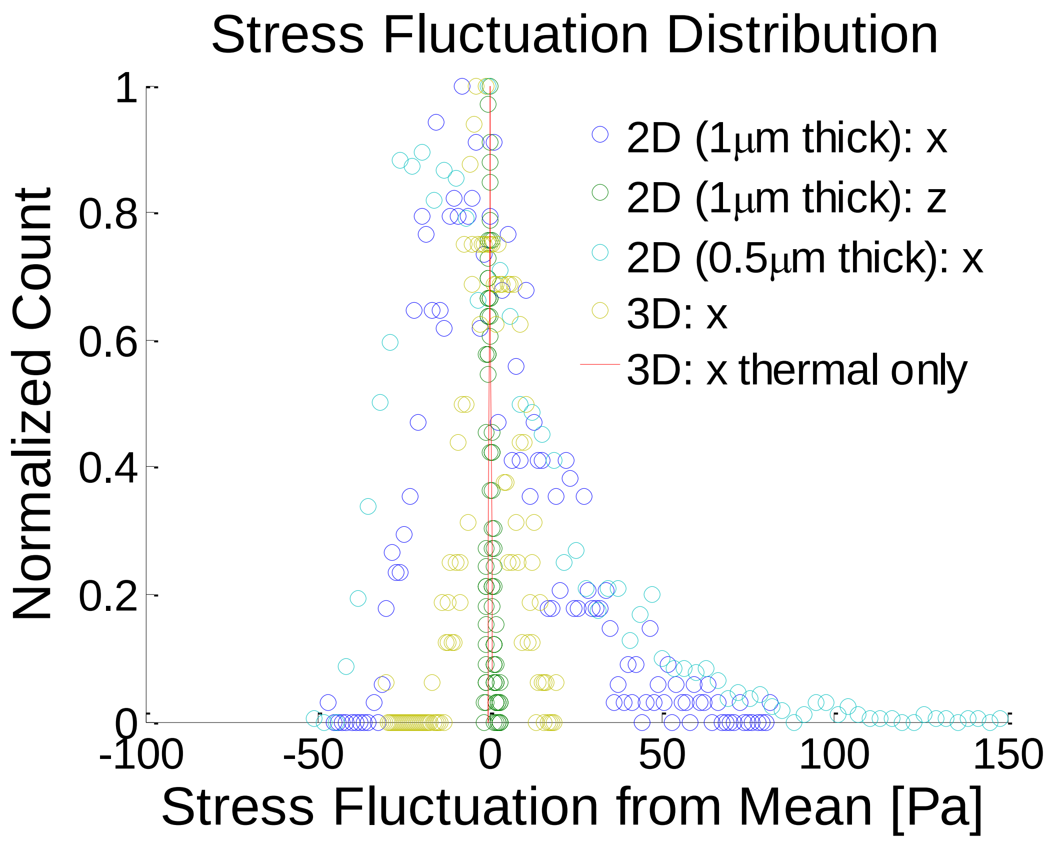

SI Figure 5: Stress fluctuation distribution from Brownian dynamics simulations. The normalized distribution of stress fluctuations from mean stress for different conditions is shown. For 2D simulations, the distribution is wider in the x (and y) direction than in the z direction, which has the fixed boundaries. Decreasing the height in 2D (z-dimension) appears to increase the width of the stress fluctuation distribution. In 3D, the distribution has a decreased width, indicating decreased stress fluctuation activity in comparison to 2D. Finally, when motors are not active and only thermal motion is present (red), the stress fluctuation magnitude is much lower.
